# Metabolic responses to high-fat diets rich in *n-3 *or *n-6 *long-chain polyunsaturated fatty acids in mice selected for either high body weight or leanness explain different health outcomes

**DOI:** 10.1186/1743-7075-8-56

**Published:** 2011-08-11

**Authors:** Karin Nuernberg, Bernhard H Breier, Shakeela N Jayasinghe, Hannes Bergmann, Nichola Thompson, Gerd Nuernberg, Dirk Dannenberger, Falk Schneider, Ulla Renne, Martina Langhammer, Korinna Huber

**Affiliations:** 1 Leibniz Institute for Farm Animal Biology, Department of Muscle Biology and Growth, 18196 Dummerstorf, W.-Stahl-Allee 2, Germany; 2 Institute of Food, Nutrition and Human Health, Massey University, Albany Campus, Private Bag 102 904, North Shore Mail Centre, Auckland, New Zealand; 3 School of Biological Sciences, Faculty of Science, University of Auckland, Auckland, New Zealand; 4 Department of Physiology, University of Veterinary Medicine Hannover, 30173 Hannover, Germany; 5 Discipline of Physiology, School of Medical Sciences, The University of Adelaide, Adelaide, Australia; 6 Leibniz Institute for Farm Animal Biology, Department of Genetic and Biometry, 18196 Dummerstorf, W.-Stahl-Allee 2, Germany; 7 Leibniz Institute for Farm Animal Biology, Department of Reproductive Biology, 18196 Dummerstorf, W.-Stahl-Allee 2, Germany

**Keywords:** polyunsaturated fatty acids, high fat diet, metabolic response, mice, selection line

## Abstract

**Background:**

Increasing evidence suggests that diets high in polyunsaturated fatty acids (PUFA) confer health benefits by improving insulin sensitivity and lipid metabolism in liver, muscle and adipose tissue.

**Methods:**

The present study investigates metabolic responses in two different lines of mice either selected for high body weight (DU6) leading to rapid obesity development, or selected for high treadmill performance (DUhTP) leading to a lean phenotype. At 29 days of age the mice were fed standard chow (7.2% fat, 25.7% protein), or a high-fat diet rich in *n*-3 PUFA (n-3 HFD, 27.7% fat, 19% protein) or a high-fat diet rich in *n*-6 PUFA (n-6 HFD, 27.7% fat, 18.6% protein) for 8 weeks. The aim of the study was to determine the effect of these PUFA-rich high-fat diets on the fatty acid profile and on the protein expression of key components of insulin signalling pathways.

**Results:**

Plasma concentrations of leptin and insulin were higher in DU6 in comparison with DUhTP mice. The high-fat diets stimulated a strong increase in leptin levels and body fat only in DU6 mice. Muscle and liver fatty acid composition were clearly changed by dietary lipid composition. In both lines of mice n-3 HFD feeding significantly reduced the hepatic insulin receptor β protein concentration which may explain decreased insulin action in liver. In contrast, protein kinase C ζ expression increased strongly in abdominal fat of n-3 HFD fed DUhTP mice, indicating enhanced insulin sensitivity in adipose tissue.

**Conclusions:**

A diet high in *n*-3 PUFA may facilitate a shift from fuel deposition in liver to fuel storage as fat in adipose tissue in mice. Tissue specific changes in insulin sensitivity may describe, at least in part, the health improving properties of dietary *n*-3 PUFA. However, important genotype-diet interactions may explain why such diets have little effect in some population groups.

## Background

Obesity has increased at an alarming rate over the past few decades, affecting a large population worldwide. Major medical complications including type 2 diabetes and cardiovascular disease are clearly linked with obesity and are increasing public health concerns [1,2]. The complex pathogenesis of obesity and type 2 diabetes is influenced by both genetic and environmental variables [3]. One of the most important contributing factors of obesity and associated metabolic complications is a diet of high caloric density which promotes excessive body weight gain [4]. The high saturated fat content of the Western diet is considered to be at least in part responsible for the perturbations in energy regulation observed in many obese individuals [1,4].

In addition to the total calorie intake from dietary fat, mounting evidence suggests that the absolute and relative intake of specific *n*-6 and *n*-3 long-chain polyunsaturated fatty acids (PUFA) contribute to the metabolic outcome [5]. Furthermore, specific dietary lipids can individually influence metabolic pathways, changing the plasma lipid profile and gene expression [6-8]. Recent studies have shown that increasing saturated fatty acids in an obesogenic high-fat diet (HFD) significantly impairs insulin sensitivity in liver, skeletal muscle, heart muscle and adipose tissue in rodents [9-15]. Furthermore, diets rich in *n*-6 PUFA may promote abdominal obesity, possibly by increasing sterol regulatory element binding protein 1c (SREBP1c) in visceral adipose tissues of rats, thereby stimulating lipogenic pathways [16]. Conversely, it has been proposed that diets rich in *n*-3 PUFA had anti-obesogenic effects and lead to a decrease in fat deposition and fasting serum triglyceride concentrations concomitant with an increase in energy expenditure and fatty acid oxidation in human and rodent studies [17,18]. Dietary lipids are also known to influence tissue fatty acid composition which is linked with changes in tissue function. For example, PUFA are important constituents of the lipid bilayer, enhancing membrane fluidity of cells and modifying cellular processes in rodents [19,20]. Therefore, dietary fatty acids play important roles in the structural composition of cells as well as in the signalling functions of specific cellular processes.

Obesity is also influenced by genetic factors. It is clear that obesity runs in families and the pattern of inheritance of obesity strongly suggests that the effect is polygenic, with each variant of many different genes making a small contribution to the outcome [21,22]. However, the strong environmental effects that are observed have led to the notion that obesity is the result of an interaction between a genetic predisposition and environmental influences, although the specific biological nature of these interactions requires further investigation. Mice from long-term selection experiments offer unique models to examine the contributions of genetic factors in determining specific dietary influences on metabolic regulation relevant to obesity development. Mice lines with biologically distinct traits, of an "*energy storage phenotype*", the high body weight line (DU6); and of an "*energy usage phenotype*", the high treadmill performance line (DUhTP), were generated by long-term selection from a common genetic pool of outbred mice [23-25]. The nature of the high body weight gain and obesity development in DU6 mice is polygenic and appears to be linked to several quantitative trait loci on distinct chromosomes contributing to higher body weight, increased fat accumulation and metabolic changes [25-27]. Although the genetic basis of leanness in DUhTP mice has not been established, their phenotypic characteristics include lower body weight, reduced food intake and smaller fat depots [23,24]. There is also expanding research evidence that selection of muscle performance is commonly based on multiple gene polymorphisms [28-30].

The aim of the present study was to investigate the fatty acid composition of tissues, and the metabolic responses to high-fat diets rich in either *n*-3 or *n*-6 PUFA in mice, with a known predisposition for high body weight or leanness. The study also investigates whether different metabolic responses may be linked to changes in insulin signalling pathways in liver, muscle and abdominal adipose tissue. Therefore, it is hypothesised that mice selected for either high body weight or leanness show different responses in lipid metabolism and alterations in insulin effectiveness. To test this hypothesis, we examined the effect of *n*-3 PUFA and *n*-6 PUFA rich high-fat diets on body weight, fatty acid composition, metabolic regulation and insulin signalling pathways in liver, muscle and adipose tissue of mice selected for either high body weight or for high running performance. The work presented here advances our understanding of the metabolic responses to high-fat diets rich in *n*-3 and *n*-6 PUFA and whether changes in insulin action and/or lipid metabolism in liver, muscle and adipose tissue may be involved. Different genetic predispositions (e.g. for leanness or obesity) could favour specific metabolic pathways in the down-stream effects on lipid or carbohydrate metabolism and influence the health outcome.

## Materials and methods

### Animals

In total 60 male mice of two different selection lines and three dietary treatments were included in this study. The selection of mice was based on traits for either high body weight or high endurance fitness and was performed at the Leibniz Institute in Dummerstorf (DU lines). The high body weight line (DU6) was selected for high body weight at day 42 (6 weeks) of life over 128 generations [25]. The high treadmill performance line (DUhTP) was selected for high treadmill performance measured by a computer-controlled treadmill at day 70 of life for 95 generations [23,24]. Thirty mice of each selected line were subdivided in 3 feeding groups with 10 mice per group. The isocaloric high-fat diets (HFD) were enriched either with *n*-3 PUFA (27% fish oil, *n*-3/*n*-6 (quotient 6.2); n-3 HFD) or with *n*-6 PUFA (27% sunflower oil, *n*-3/*n*-6 (quotient 0.0062); n-6 HFD). The control group was fed with standard mice chow (Altromin, Lage Germany; 7% fat; standard chow). The composition of the different diets is presented in Table 1. The control diet had a higher crude protein content compared to the high-fat diets. Each animal was kept individually in a cage equipped with wooden shavings and with free access to water. From birth until the 28^th ^day of life all animals were fed with the standard chow diet. From the 29^th ^until the 87^th ^day, animals were either allocated to one of the high-fat diets or were kept continuously on the standard chow. All diets were provided *ad libitum*. Body weight was recorded weekly until the end of the experiment. Mean food intake was determined weekly for each animal. Daily food intake, food intake per g metabolic body weight (bwt^0.75^) and daily calorie intake per g bwt^0.75 ^were calculated for all experimental groups. All animal procedures were approved by the local animal welfare authority (Reg.-No. LALLF M-V/TSD/7221.3-2.1-011/06).

**Table 1:** Composition of the standard chow and the PUFA-rich high-fat diets

	n-3 HFD	n-6 HFD	Standard chow
Crude protein (%/DM)	19·0	18·6	25·7
Crude fat (%/DM)	27·4	27·7	7·2
Crude ash (%/DM)	5·1	5·0	8·1
Metabolisable energy (MJ/DM)	20·5	20·6	15·9
Fatty acid composition (wt %)			
Myristic acid (C14:0)	6·3	0·4	0·8
Palmitic acid (C16:0)	14·7	6·1	13·2
Stearic acid (C18:0)	2·9	3·7	2·8
Oleic acid (C18:1*cis*-9)	14·3	22·5	20·6
Linoleic acid (C18:2*n*-6)	4·9	64·4	54·1
α Linolenic acid (C18:3*n*-3)	7·0	0·1	4·5
Arachidonic acid (C20:4*n*-6)	0·6	n.a.	n.a.
Eicosapentaenoic acid (C20:5*n*-3)	12·3	0·2	0·2
Docosapentaenoic acid (C22:5*n*-3)	2·1	0·01	0·05
Docosahexaenoic acid (C22:6*n*-3)	16·0	0	0·04
Σ *n*-3 FA	39·9	0·4	4·9
Σ *n*-6 FA	6·4	64·4	54·2

Fatty acid content is given in weight (wt) %; n.a. not analysed

DM-dry matter; PUFA-polyunsaturated fatty acids; FA-fatty acids; HFD-high fat diet.

### Tissue and blood sample collection

At the end of the study at day 87, the animals were killed by cervical dislocation and trunk blood samples were collected after decapitation. Liver, *quadriceps femoris *muscle and abdominal adipose tissue were dissected immediately and were weighed, snap frozen in liquid nitrogen and stored at -80°C until analysis. Analyses were performed within one year after sampling. EDTA blood was centrifuged at 2750 g at 4°C for 5 minutes. Plasma was removed, centrifuged again and stored at -20°C for future analysis.

### Plasma analyses

Plasma leptin was measured by a commercial sandwich-type ELISA (DSL, Webster, Texas, U.S.A.). The sensitivity (minimum detection limit) was 0.3 ng/ml. The coefficient of intra-assay variation was 5.9%, the coefficient of inter-assay variation was 7.2%. The plasma insulin levels were measured by a commercial radioimmunoassay (RIA) (sensitive rat RIA, Linco, St. Charles, Missouri, U.S.A.). The coefficient of intra-assay variation was 5.5%, the coefficient of inter-assay variation was 8.6%. Total cholesterol, free fatty acids and glucose concentrations were analysed using a COBAS MIRA Chemistry Analyzer (Roche, Switzerland) and respective test kits. Total cholesterol was determined by colorimetric measurements with end point detection of cholesterol oxidase, phenol, aminoantipyrin (CHOD-PAP) (Labor und Technik Lehmann, Berlin, Germany). Free fatty acids were determined by colorimetric and enzymatic measurements (RANDOX, Crumlin, UK). Plasma glucose was determined by colorimetric measurements of glucose-oxidase, phenol, aminoantipyrin (GOD-PAP) (Labor und Technik Lehmann, Berlin, Germany).

### Determination of fatty acid composition

Samples of livers (0.4-0.6 g) and *quadriceps femoris *muscles (0.04-0.07 g) were thawed at 4°C. After homogenisation (Ultra Turrax, IKA Staufen, Germany; T25, 3 × 15 sec, 12,000 rpm) and adding C19:0 as an internal standard, the total lipids were extracted in duplicates with chloroform/methanol (2:1, v/v) at room temperature. All the solvents contained 0.005% (w/v) of t-butylhydroxytoluene in order to avoid oxidation of PUFA. The extraction mixture was stored at 5°C for 18 h in the dark and subsequently washed with 0.02% aqueous CaCl_2_. The organic phase was dried with Na_2_SO_4 _and K_2_CO_3 _(10:1, wt/wt) and the solvent was subsequently removed under nitrogen at room temperature. The lipid extracts were re-dissolved in toluene and 3 mg (muscle) to 20 mg (liver) was used for methyl ester preparation. Fatty acids of liver and muscle were determined by gas chromatography as previously described by Dance *et al*. [31]. Delta-9 desaturase index was calculated by the following equation ((C14:1+C16:1+C18:1+C17:1)/(C14:1+C16:1+C18:1+C17:1+C14:0+C16:0+C17:0+ C18:0)) × 100 = Δ9. This index largely reflects desaturase activity.

### Tissue sample preparation

Liver, *quadriceps femoris *muscle and abdominal adipose tissue samples were homogenised in ice-cold RIPA lysis buffer (Triton X-100, sodium dodecyl sulphate, sodium chloride, Tris-HCl, deoxycholic acid, sodium orthovanadate, and Complete Mini EDTA-free protease inhibitors from Roche, Diagnostics) and stored at -80°C until analysis. Protein concentrations of the liver and muscle homogenates were determined using a Bradford assay.

*Western blotting *was performed as described earlier [32]. In short, SDS-PAGE was performed using 8% separation gels. Proteins were transferred onto nitrocellulose membranes by tank blotting, blocked with fat-free milk (5%) overnight (or for 2 h at RT for protein kinase C zeta (PKC ζ) detection) and exposed to the respective primary antibody for 1 h at RT (insulin receptor β subunit (IR β; Santa Cruz Biotechnology Inc, Santa Cruz, CA) and phosphoinositol-3-kinase (PI3K; Upstate Biotechnology, Lake Placid, NY) or overnight for PKC ζ (Santa Cruz Biotechnology Inc). Specific antibodies were detected by secondary antibodies conjugated to horse radish peroxidase (1 h at RT; Sigma-Aldrich) and chemiluminescence was used for protein visualisation (Pierce). Specific protein expression was quantified densitometrically using Quantity One software (Bio-Rad). Intensity of signals between different blots was compared by using one or two reference samples on each blot.

### Statistical analyses

**Figure 1:**
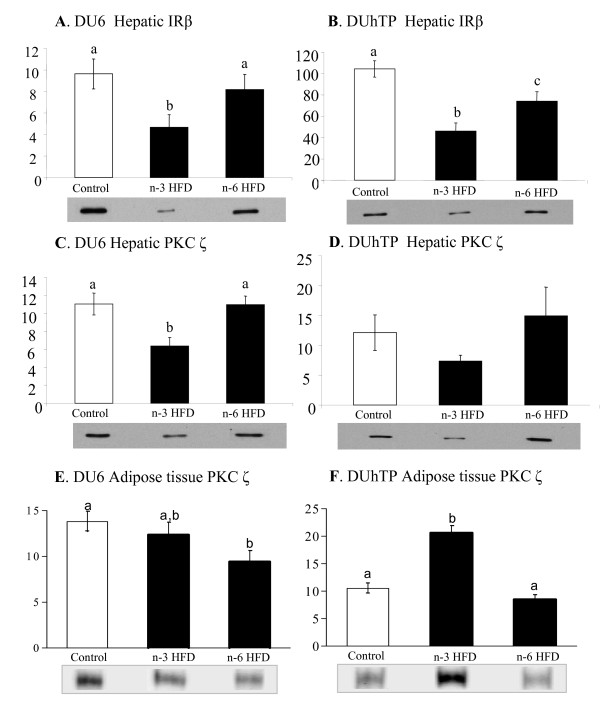
**Effects of *n-3 *and *n-6 *high-fat diets on expression of insulin receptor β protein in liver (A, B) and protein kinase C ζ protein in liver (C, D) and abdominal adipose tissue (E, F) in mice with distinct biologically traits, of an "*energy storage phenotype*", the DU6 mice (high body weight line (A, C, E) and an "*energy usage phenotype*", the DUhTP mice (high treadmill performance line) (B, D, F)**. Trace quantity data are means ± SE, n = 10/group; lower case letter mark significant differences p < 0.05.

For the statistical analyses of the of insulin signalling parameters a One-Way Analysis of Variance (effect of diet within lines) was used (SAS^© ^Systems, Release 8.2, SAS Institute Inc., Cary, NC (SAS)). Protein expression was determined for each mice line separately comparing the different feeding groups. Statistical analysis for all the other traits was performed using the least-squares method and the GLM procedures of SAS. The following model was employed for the analysis of variance with the fix factors feeding (D) and selection (S): Y_ijk _= μ+ D_i _+ P_j _+ D_i _× S_j _+ E_ijk _(with μ = overall mean; D_i _= diet effect (i = 3); S_j _= effect of selection (j = 2); D_i _× P_j _= Interaction between diet and selection; E_ijk _= residual error). For the following variables, plasma leptin, C18:2*n*-6, C20:5*n*-3, C22:5*n*-3, C22:6n-3 and the ratio *n*-6/*n*-3 in liver and C18:3*n*-3, C20:5*n*-3 and the ratio of *n*-6/*n*-3 in muscle with different variances in diet groups, the SAS proc mixed program was used to model these different variances (by repeated statement and option group = diet). All Tables and Figure 1 contain the least squares mean (LSM) and the standard error (SE). All statistical tests (Tukey-Kramer) of LSM were performed for a significance level of p ≤ 0.05.

## Results

The daily food intake was significantly different in the selection lines and clearly influenced by the different diets. Based on the long-term selection process described above, the DU6 mice showed a rapid growth trajectory during early postnatal life (overall difference in daily weight gain DU6 versus DUhTP, factor genetic line p < 0.0001, factor diet p < 0.001, interaction p < 0.01; (mean daily weight gain in g/d DU6-standard chow 1.04 ± 0.03, n-3 HFD 1.21 ± 0.02, n-6 HFD 1.23 ± 0.02 (standard chow versus HFD p < 0.001) DUhTP-standard chow 0.34 ± 0.01, n-3 HFD 0.40 ± 0.02, n-6 HFD 0.37 ± 0.01 (standard chow versus n-3 HFD p < 0.05)) and were much heavier throughout the study (Table 2). Consequently, the DU6 mice consumed twice the amount of food than the DUhTP mice (Table 2). Feeding the HFD diets led to a decrease in the total amount of food (in grams) consumed in both mice lines compared to standard chow feeding. However, the relative amounts of food consumed, adjusted for the metabolic body weight (bwt^0.75^) of the animals, was comparable between the DU6 and DUhTP mice, there was no effect of selection line. The daily calorie intake relative to bwt^0.75 ^further revealed that all groups consumed very similar quantities of energy with only slightly higher calorie intake in the DUhTP mice fed the n-3 HFD (Table 2).

**Table 2:** Effect of diet on body composition, daily food and energy intake of different mice lines

	DU6			DUhTP		
	
	Standard chow	n-3 HFD	n-6 HFD	Standard chow	n-3 HFD	n-6 HFD
	LSM ± SE	LSM ± SE	LSM ± SE	LSM ± SE	LSM ± SE	LSM ± SE
Body weight (bwt) at 87^th ^day (g)	84·0 ± 1·6^a, A^	96·2 ± 1·7^b, A^	98·4 ± 1·5^b, A^	28·.8 ± 1·6^a, B^	35·4 ± 1·5^b, B^	32·8 ± 1·6^B^
Daily food intake (g/d)	11·4 ± 0·2^a, A^	8·8 ± 0·2^b, A^	8·8 ± 0·2^b, A^	5·2 ± 0·2^a, B^	4·8 ± 0·2^a, b, B^	4·1 ± 0·2^b, B^
Daily food intake (g/bwt^0.75^)	0·41 ± 0·02^a^	0·29 ± 0·02^b^	0·28 ± 0·02^b^	0·42 ± 0·02^a^	0·33 ± 0·02^b^	0·30 ± 0·02^b^
Daily calorie intake (calorie/bwt^0.75^)	1·24 ± 0·12	1·28 ± 0·12	1·24 ± 0·08	1·25 ± 0·28^a^	1·49 ± 0·13^b^	1·32 ± 0·05^a, b^
Liver (g)	3·7 ± 0·1^a, A^	4·1 ± 0·1^b, A^	3·5 ± 0·1^a, A^	1·1 ± 0·1^a, B^	1·5 ± 0·1^b, B^	1·2 ± 0·1^a, B^
Abdominal fat (g)	2·6 ± 0·3^a, A^	4·6 ± 0·3^b, A^	5·2 ± 0·2^b, A^	0·3 ± 0.3^B^	1·0 ± 0.2^B^	0·7 ± 0·3^B^
Perirenal fat (g)	2·0 ± 0·2^a, A^	3·4 ± 0·2^b, A^	3·8 ± 0·2^b, A^	0·2 ± 0·2^B^	0·5 ± 0·2^B^	0·4 ± 0·2^B^
Brown fat (g)	0·20 ± 0·016^a, A^	0·26 ± 0·017^b, A^	0·25 ± 0·015^b, A^	0·07 ± 0·016^B^	0·13 ± 0·015^B^	0·098 ± 0·016^B^
Liver fat (%)	7.1 ± 0·9^a^	8·0 ± 1·0^a, b^	10·2 ± 0·9^b, A^	6·7 ± 0·9	6·8 ± 0·9	8·7 ± 0·9^B^
Muscle fat (%)	2·6 ± 0.2	3·5 ± 0·2	2·9 ± ·02	2·9 ± 0·2	3·1 ± 0·1	3·0 ± 0·2

DU6-mouse line selected for high body weight; DUhTP-mouse line selected for high treadmill performance

n-3 and n-6 HFD-high fat diet enriched with either *n*-3 or *n*-6 polyunsaturated fatty acids

LSM-Least square means of 8-10 mice per group, SE-Standard error, bwt-body weight, bwt^0.75^- metabolic body weight

a, b-significant effect of diet at p ≤ 0.05 within the selected line

A, B -significant effect of selection line at p ≤ 0.05 within the feeding group.

### Lipid metabolism

Body fat deposition was influenced by selection line and by the specific diet. The DU6 mice deposited significantly more abdominal, perirenal and brown fat compared to DUhTP animals (Table 2). Consequently, the body weight at day 87 (end of the experiment) of DU6 mice was significant higher than the body weight of DUhTP mice in all feeding groups. Within the groups of DU6 mice, feeding either of the HFD resulted in a further increase in the weight of abdominal, perirenal and brown adipose tissues (Table 2). In contrast, feeding a HFD to DUhTP mice did not result in significant changes in fat deposition with abdominal adipose tissue weight reaching a small trend towards increased levels (p = 0.06) in the n-3 HFD-fed animals compared to the standard chow-fed group (Table 2). Plasma leptin was measured as a blood marker of total adiposity. The larger adipose tissue mass in DU6 mice was reflected in leptin levels which were much higher in DU6 animals than in DUhTP animals (Table 3). Both HFD diets further increased plasma leptin concentrations in the DU6 mice compared to DU6 fed standard chow. The n-6 HFD had no effect on plasma leptin concentration in DUhTP animals while n-3 HFD increased plasma leptin concentrations slightly. Plasma FFA levels were higher in DU6 mice but unaffected by the HFD diets while total cholesterol was comparable in both selection lines. However, n-3 HFD decreased the cholesterol level significantly in DUhTP mice compared to the chow diet and the n-6 HFD groups (Table 3). Fat deposition in liver and muscle was not influenced by selection line or by feeding HFD except in n-6 HFD-fed DU6 mice, showing an increase in hepatic fat content (Table 2). However, n-3 HFD increased liver weight in both, DU6 and DUhTP (Table 2).

**Table 3:** Effect of diet on blood parameter of different mice lines

	DU6			DUhTP		
	
	Standard chow	n-3 HFD	n-6 HFD	Standard chow	n-3 HFD	n-6 HFD
	LSM ± SE	LSM ± SE	LSM ± SE	LSM ± SE	LSM ± SE	LSM ± SE
Leptin (ng/ml)	9·8 ± 3·1^a, A^	27·9 ± 2·6^b, A^	27·2 ± 5·5^b, A^	0·8 ± 0·3^a, B^	2·5 ± 0·6^b, B^	1·1 ± 0·4^a, b, B^
Cholesterol (mM)	4·7 ± 0·8	3·5 ± 0·9	5·2 ± 0·8	6·4 ± 0·9^a^	4·0 ± 0·8^b^	6·5 ± 0·8^a^
FFA (mM)	1·2 ± 0·1^A^	1·2 ± 0·1^A^	1·4 ± 0·1^A^	0·8 ± 0·1^B^	0·5 ± 0·1^B^	0·8 ± 0·1^B^
Insulin (ng/ml)	1·38 ± 0·3^a, b, A^	0·98 ± 0·3^a^	1·81 ± 0·3^b, A^	0·23 ± 0·3^B^	0·21 ± 0·3	0·19 ± 0·3^B^
Glucose (mM)	8·9 ± 0·8^a^	11·0 ± 0·8^b, A^	11·2 ± 0·8^b, A^	8·9 ± 0·8	8·9 ± 0·8^B^	7·9 ± 0·8^B^

DU6-mouse line selected for high body weight; DUhTP-mouse line selected for high treadmill performance

n-3 and n-6 HFD-high fat diet enriched with either *n*-3 or *n*-6 polyunsaturated fatty acids

LSM-Least square means of 7-10 mice per group, SE-Standard error; FFA-free fatty acids;

a, b-significant effect of diet at p ≤ 0.05 within the selected line

A, B-significant effect of selection line at p ≤ 0.05 within the feeding group.

### Glucose metabolism

Plasma glucose levels were similar in DU6 and DUhTP mice fed with standard chow. However, in DU6 mice both HFD diets increased glucose concentrations significantly. This was accompanied by an increase in plasma insulin but only in the DU6 mice fed on n-6 HFD. Insulin levels of DUhTP mice were generally lower and not influenced by HFD diets (Table 3). Expression of key proteins of insulin signalling in liver and abdominal adipose tissue was clearly influenced by HFD diets, but the effects differed in both selection lines. Hepatic IRβ expression was reduced in both, the DU6 and DUhTP animals fed on n-3 HFD. Furthermore, hepatic IRβ expression was also reduced in DUhTP animals fed on n-6 HFD but this reduction was less pronounced than in DUhTP mice fed on n-3 HFD (Figure 1). Hepatic p58 subunit of PI3K was increased only in DU6 mice fed on n-6 HFD compared to DU6 fed on n-3 HFD (160.3 ± 17.1 versus 90.5 ± 19.7, p < 0.001). Hepatic PKC ζ was significantly reduced only in DU6 mice fed on n-3 HFD while adipose tissue PKC ζ was reduced in DU6 mice fed on n-6 HFD. In DUhTP mice fed n-3 HFD a dramatic increase of PKC ζ protein expression was observed in adipose tissue while the n-6 HFD had no effect (Figure 1). In muscle (*M. quadriceps femoris*), high-fat feeding did not influence expression levels of IRβ, PI3-kinase and PKC ζ in both selected lines (data not shown).

### Lipids in liver

Analyses of liver lipids are shown in table 4. Differences based on selection line were mainly observed in mice fed n-6 HFD; there were fewer selection line effects with the standard chow diet and no selection line effects with n-3 HFD feeding. DU6 mice fed n-6 HFD synthesised significantly more palmitic acid (C16:0) and less stearic acid (C18:0) and arachidonic acid (C20:4*n*-6; AA) compared to DUhTP mice. Furthermore, DU6 mice fed n-6 HFD or standard chow accumulated more oleic acid (C18:1*cis*-9). As expected, n-3 HFD feeding of both selection lines massively increased the amount of eicosapentaenoic acid (C20:5*n*-3; EPA), docosapentaenoic acid (C22:5*n*-3; DPA) and docosahexaenoic acid (C22:6*n*-3; DHA) and resulted in a lower deposition of AA.

**Table 4:** Effect of diet on fatty acid composition of liver of different mice lines

	DU6			DUhTP		
	
	Standard chow	n-3 HFD	n-6 HFD	Standard chow	n-3 HFD	n-6 HFD
	LSM ± SE	LSM ± SE	LSM ± SE	LSM ± SE	LSM ± SE	LSM ± SE
						
SFA^+^	2420 ± 222	2794 ± 251	2981 ± 210	1889 ± 222	2520 ± 210	2224 ± 222
C14:0	23·2 ± 8^a^	73·1 ± 9^b^	41·2 ± 7^a, b^	20·0 ± 8^a^	77·7 ± 7^b^	23·4 ± 8^a^
C16:0	1888 ± 186	2057 ± 211	2292 ± 177^A^	1335 ± 186	1721 ± 177	1351 ± 186^B^
C18:0	435·3 ± 33	560·1 ± 38	547·5 ± 32^A^	436·3 ± 33^a^	589·5 ± 32^b^	733·6 ± 33^c, B^
MUFA^#^	2074 ± 225^A^	1567 ± 256	2544 ± 214^A^	1006 ± 225^B^	1473 ± 214	1547 ± 226^B^
C16:1*cis*-9	218·4 ± 26	247·8 ± 29	143·5 ± 24	110·1 ± 26^a^	244·6 ± 24^b^	89·9 ± 25^a^
C18:1*cis-*9	1636 ± 184^a, b, A^	1178 ± 209^a^	2225 ± 175^b, A^	793 ± 185^B^	1044 ± 175	1355 ± 185^B^
∑*n*-6 FA^d^	2706 ± 438^a^	754·6 ± 497^b^	6135 ± 416^c^	2283 ± 438^a^	759·7 ± 416^b^	5876 ± 438^a^
C18:2*n*-6	1941 ± 269^a^	474·8 ± 47^b^	5003 ± 575^c^	1686 ± 349^a^	478 ± 39^b^	4605 ± 587^c^
C20:4*n*-6	557·7 ± 38^a^	226·8 ± 43^b^	727·2 ± 37^cA^	479·3 ± 39^a^	236·9 ± 37^b^	902·9 ± 39^cB^
∑*n*-3 FA^e^	505·0 ± 144^a^	2385 ± 163^b^	237·9 ± 136^a^	556·6 ± 144^a^	2197 ± 136^b^	110·1 ± 144^a^
C18:3*n-*3	70·4 ± 11^a^	93·3 ± 13^a^	13·3 ± 11^b^	63·2 ± 11^a^	85·1 ± 11^a^	8·4 ± 2^b^
C20:5*n-*3	15·6 ± 3^a^	476·6 ± 64^b^	10·0 ± 4^a^	13·2 ± 3^a^	400·9 ± 53^b^	3·7 ± 4^a^
C22:5*n-*3	22·2 ± 4^a^	133·8 ± 20^b^	11·3 ± 5^a^	33·0 ± 4^a^	114·0 ± 17^b^	20·1 ± 6^a^
C22:6*n-*3	387·6 ± 19^a^	1634 ± 180^b^	191·3 ± 32^a^	439·3 ± 19^a^	1556 ± 150^b^	73·4 ± 34^a^
PUFA	3219 ± 476^a^	3144 ± 539^a^	6404 ± 451^b^	2844 ± 476^a^	2962 ± 451^a^	5999 ± 476^b^
Δ9 Desaturase index^f^	58·8 ± 2^a, A^	34·4 ± 3^b^	44·5 ± 2^c^	27·0 ± 2^a, B^	34·2 ± 2^a, b^	39·3 ± 2^b^
*n*-6/*n*-3 ratio	5·3 ± 0·3^a^	0·3 ± 0·02^b^	37·6 ± 6^c, A^	4·0 ± 0·3^a^	0·4 ± 0·02^b^	52·3 ± 6^c, B^

DU6-mouse line selected for high body weight; DUhTP-mouse line selected for high treadmill performance n-3 and n-6 HFD -high fat diet enriched with either *n*-3 or *n*-6 polyunsaturated fatty acids

LSM-Least square means, SE-standard error, fatty acid concentrations in mg/100 g fresh tissue;

MUFA-monounsaturated fatty acids; SFA-saturated fatty acids; PUFA-polyunsaturated fatty acids; FA-fatty acids;

a, b, c-significant effect of diet at p ≤ 0.05 within the selected line

A, B-significant effect of selection line at p ≤ 0.05 within the feeding group

^+ ^Sum of saturated fatty acids (C10:0+C11:0+C12:0+C13:0+C14:0+C15:0+C16:0+C17:0+C18:0+C20:0+C21:0+C22:0+C23:0+ C24:0)

^# ^Sum of MUFA (C14:1+C16:1+C17:1+∑ C18:1 *trans *isomers+C18:1*cis-*9+C18:1*cis-*11+C22:1+C24:1)

^d ^Sum of *n*-6 fatty acids (C18:3*n*-6+ C22:4*n*-6+ C20:3*n*-6+ C18:2*n*-6+ C20:4*n*-6)

^e ^Sum of *n*-3 fatty acids (C20:3*n*-3+ C22:6*n*-3+ C22:5*n*-3+ C20:5*n*-3+ C18:4*n*-3+ C18:3*n*-3)

^f ^Δ9 Desaturase index = ((C14:1+C16:1+C18:1+C17:1)/(C14:1+C16:1+C18:1+C17:1+C14:0+C16:0+C17:0+ C18:0))*100.

Comparable to observations in muscle tissue, there were high amounts of myristic acid (C14:0) in liver of both mice lines when fed n-3 HFD. Concentration of oleic acid was lowest in DU6 mice fed n-3 HFD. Correspondingly, the calculated Δ^9 ^desaturase (SCD) index was low in DU6 mice fed n-3 HFD compared to standard chow and n-6 HFD fed mice. Interestingly, in DUhTP mice fed standard chow, the SCD index was lower compared to that in DU6 mice, indicating a difference determined by selection. However, the SCD index was similar when DUhTP mice were fed n-3 and n-6 HFD, respectively. Accumulation of linoleic acid (C18:2*n*-6; LA) and AA was highest in the liver of n-6 HFD-fed mice of both selection lines. Feeding n-6 HFD increased the ratio of *n*-6/*n*-3 fatty acids in the liver fat of both selection lines, and this effect was strongest in DUhTP mice.

### Lipids in quadriceps femoris muscle

The lipid concentrations of muscle tissue are presented in table 5. The fatty acid composition of skeletal muscle showed clear differences across the selection lines, but these patterns were distinct to those found in liver. Intriguingly, the DUhTP mice fed standard chow showed higher levels of DPA and DHA deposition in muscle compared to the DU6 animals. Concentrations of saturated fatty acids (SFA), monounsaturated fatty acids (MUFA), α-linolenic acid (C18:3*n*-3; ALA) and EPA were increased in muscle of DU6 mice compared to DUhTP mice when fed n-3 HFD. The DU6 mice showed higher concentrations of LA and AA when fed n-6 HFD resulting in a comparable *n*-6/*n*-3 ratio in both selection lines. The DU6 mice showed an increase in all SFA in muscle tissue in response to n-3 HFD compared to n-6 HFD and standard chow. This was not observed in DUhTP with the exception of the significant increase of myristic acid in both mice lines. Other responses to n-3 and n-6 HFD were quite similar between both selected lines. A summary of the responses to the HFD in both selection lines is presented in table 6.

**Table 5:** Effect of diet on fatty acid composition of *quadriceps femoris *muscle of different mice line

	DU6			DUhTP		
	
	Standard chow	n-3 HFD	n-6 HFD	Standard chow	n-3 HFD	n-6 HFD
	LSM ± SE	LSM ± SE	LSM ± SE	LSM ± SE	LSM ± SE	LSM ± SE
SFA^+^	365·5 ± 45^a^	697·2 ± 48^b, A^	376·6 ± 43^a^	327·0 ± 45	325·1 ± 43^B^	218·2 ± 45
C14:0	11·21 ± 4^a^	62·31 ± 4^b, A^	9·58 ± 4^a^	6·5 ± 4^a^	24·1 ± 4^b, B^	3·9 ± 4^a^
C16:0	265·9 ± 34^a^	488·5 ± 36^b, A^	248·4 ± 32^a^	199·3 ± 34	210·0 ± 32^B^	120·2 ± 34
C18:0	62·4 ± 6^a^	98·4 ± 6^bA^	91·2 ± 6^a, b^	85·2 ± 6^a^	57·7 ± 6^b B^	68·9 ± 6^a, b^
MUFA^#^	225·6 ± 50^a^	568·2 ± 53^b, A^	375·7 ± 47	180·0 ± 50	280·7 ± 47^B^	183·1 ± 50
C16:1*cis*-9	70·4 ± 10^a, b, A^	96·6 ± 10^a, A^	36·7 ± 9^b^	25·4 ± 10^B^	41·6 ± 9^B^	14·1 ± 10
C18:1*cis-*9	206·7 ± 33	320·8 ± 35^A^	296·4 ± 31^A^	116·8 ± 33	149·5 ± 31^B^	145·8 ± 33^B^
∑*n*-6 FA^d^	345·0 ± 44^a^	141·6 ± 47^b^	800·6 ± 42^cA^	302·5 ± 44^a^	70·6 ± 42^b^	445·0 ± 44^a, B^
C18:2*n*-6	225·6 ± 42^a^	100·9 ± 44^a^	653·3 ± 40^b A^	167·5 ± 42^a, B^	43·2 ± 40^a^	318·7 ± 42^bB^
C20:4*n*-6	98·2 ± 5^a^	25·4 ± 5^b^	112·7 ± 5^a, A^	108·4 ± 5^a^	20·4 ± 5^b^	91·2 ± 5^aB^
∑*n*-3 FA^e^	99·5 ± 12^a, A^	325·0 ± 13^b, A^	37·9 ± 12^c^	171·8 ± 12^a, B^	270·6 ± 12^b, B^	25·7 ± 12^c^
C18:3*n-*3	9·4 ± 1·6^a^	48·0 ± 5^b, A^	2·3 ± 0·3^a^	5·7 ± 1·6	6·8 ± 5^B^	1·3 ± 0·4
C20:5*n-*3	0·9 ± 0·1^a^	50·5 ± 0·5^b, A^	1·0 ± 0·2^a, A^	1·3 ± 0·1^a^	26·4 ± 4^bB^	0·1 ± 0·2^c, B^
C22:5*n-*3	7·9 ± 1^a, A^	23·1 ± 1^b^	3·1 ± 1^a^	22·3 ± 1^a, B^	20·5 ± 1^a^	2·7 ± 1^b^
C22:6*n-*3	80·6 ± 9^a, A^	201·2 ± 9^b^	30·7 ± 8^c^	142·1 ± 8^a, B^	215·6 ± 8^b^	21·3 ± 9^c^
PUFA	445·2 ± 50^a^	468·8 ± 53^a^	839·6 ± 48^b, A^	475·0 ± 50	342·0 ± 47	471·7 ± 50^B^
Δ9 Desaturase index^f^	43·4 ± 2^a, b^	41·2 ± 2^a^	48·5 ± 2^b, A^	33·7 ± 2^a^	42·5 ± 2^b^	45·0 ± 2^b, B^
*n*-6/*n*-3 ratio	3·4 ± 0·2^a, A^	0·40 ± 0·02^b, A^	23·8 ± 2·6^c^	1·9 ± 0·2^a, B^	0·3 ± 0·02^b, B^	17·6 ± 2·8^c^

DU6-mouse line selected for high body weight; DUhTP-mouse line selected for high treadmill performance

n-3 and n-6 HFD-high fat diet enriched with either *n*-3 or *n*-6 polyunsaturated fatty acids

LSM-Least square means, SE-Standard error, fatty acid concentrations in mg/100 g fresh tissue;

MUFA-monounsaturated fatty acids; SFA-saturated fatty acids; PUFA-polyunsaturated fatty acids; FA-fatty acids;

a, b, c-significant effect of diet at p ≤ 0.05 within the selected line,

A, B -significant effect of selection line at p ≤ 0.05 within the feeding group; all interaction DxL were significant at p ≤ 0.05;

^+ ^Sum of saturated fatty acids: (C10:0+C11:0+C12:0+C13:0+C14:0+C15:0+C16:0+C17:0+C18:0+C20:0+C21:0+C22:0+C23:0+ C24:0)

^# ^Sum of MUFA (C14:1+C16:1+C17:1+∑ C18:1 *trans *isomers+C18:1*cis-*9+C18:1*cis-*11+C22:1+C24:1)

^d ^Sum of *n*-6 FA (C18:3*n*-6+ C22:4*n*-6+ C20:3*n*-6+ C18:2*n*-6+ C20:4*n*-6)

^e ^Sum of *n*-3 FA (C20:3*n*-3+ C22:6*n*-3+ C22:5*n*-3+ C20:5*n*-3+ C18:4*n*-3+ C18:3*n*-3)

^f ^Δ9 Desaturase index = ((C14:1+C16:1+C18:1+C17:1)/(C14:1+C16:1+C18:1+C17:1+C14:0+C16:0+C17:0+ C18:0))*100.

**Table 6:** Summary of major effects of n-3 and n-6 HFD on lipid and glucose metabolism

	DU6		DUhTP			DU6		DUhTP	
			
Lipid metabolism	n-3 HFD	n-6 HFD	n-3 HFD	n-6 HFD	Glucose metabolism	n-3 HFD	n-6 HFD	n-3 HFD	n-6 HFD
Leptin	↑	↑	↑	-	Insulin	-	(↑)	-	-
FFA	-	-	-	-	Glucose	↑	↑	-	-
Cholesterol	-	-	↓	-	Liver IRβ	↓	-	↓	↓
Fat deposition	↑	↑	(↑)	-	Liver PI3K	-	-	-	-
Body weight	↑	↑	↑	-	Liver PKCζ	↓	-	-	-
Liver weight	↑	-	↑	-	Muscle IRβ	-	-	-	-
Liver fat	-	↑	-	-	Muscle PKCζ	-	-	-	-
					Muscle PI3K	-	-	-	-
					Adipose PKCζ	-	↓	↑	-

**Fatty acid composition** **liver**					**Fatty acid composition** **muscle**				
SFA	-	-	-	-	SFA	↑	-	-	-
Myristic acid	↑	-	↑	-	Myristic acid	↑	-	↑	-
Palmitic acid	-	-	-	-	Palmitic acid	↑	-	-	-
Stearic acid	-	-	↑	↑	Stearic acid	↑	-	↓	-
Oleic acid	-	(↑)	-	-	Oleic acid	-	-	-	-
MUFA	-	-	-	-	MUFA	↑	-	-	-
PUFA	-	↑	-	↑	PUFA	-	↑	-	-
*n*-3 PUFA	↑	-	↑	-	*n*-3 PUFA	↑	↓	↑	↓
*n*-6 PUFA	↓	↑	↓	-	*n*-6 PUFA	↓	↑	↓	-

DU6-mouse line selected for high body weight; DUhTP-mouse line selected for high treadmill performance

n-3 and n-6 HFD-high fat diet enriched with either *n*-3 or *n*-6 polyunsaturated fatty acids

FFA-free fatty acids; IR β-Insulin receptor β subunit; PI3K-phosphatidyl inositol-3 kinase; PKC ζ-atypical protein kinase ζ; PUFA-polyunsaturated fatty acids; MUFA-monounsaturated fatty acids

- no change; ↑ increase compared to respective standard chow fed group; ↓ decrease compared to respective standard chow group; (↓)(↑) tendency to decrease/increase.

## Discussion

The world-wide epidemic of obesity is due to complex, multifactorial changes in lifestyle and environmental conditions. In addition to an excess of calorie intake and composition of the diet, genetically determined pathways of energy storage and utilisation contribute to the development of obesity. The role of genetic factors in the pathogenesis of obesity is undeniable with familial clusters giving individuals in our modern "obesogenic" environment markedly different risks of becoming obese [21,22]. The present study investigates the fatty acid composition of tissues and the metabolic responses to high-fat diets rich in either *n*-3 or *n*-6 PUFA in mice, selected for high body weight (DU6), leading to rapid obesity development, or selected for high treadmill performance, leading to a lean phenotype. This study also investigates whether different metabolic responses may be linked to changes in insulin signalling pathways in liver, muscle and adipose tissue. The mouse lines used in the present study reflect the range of genetic predisposition to obesity development commonly observed under physiological conditions [26].

It is most likely that selection of these mouse lines produced metabolic phenotypes by polygenic variants of genes as described in human populations [33]. The mice lines used in this study reveal distinct metabolic phenotypes. The DU6 mice display an "*energy storage phenotype" *that is characterised by high body weight, increased food intake, larger fat depots, higher liver weight and higher plasma FFA, leptin and insulin levels. In contrast, the DUhTP mice display an "*energy usage phenotype" *that is characterised by lower body weight, less food intake, smaller fat depots, lower liver weight and lower plasma FFA, leptin and insulin levels. The findings of the present study also suggest that there is a strong genetic contribution to the pattern of fatty acid composition of body tissues. The selection lines showed clear differences in the fatty acid composition of liver and muscle even under standard chow feeding conditions. These data are supported by studies in pigs which showed a significant heritability of fatty acid composition in muscle, especially for the concentrations of *n*-3 and *n*-6 PUFA [34]. Interestingly, in liver, the amount of oleic acid was lower in DUhTP mice in comparison with DU6 mice which may be related to the reduced capacity to synthesise oleic acid from stearic acid as indicated by the decrease in delta-9 desaturase index. In muscle, palmitoleic acid content was also lower in DUhTP. This difference in the SCD index between selection lines appears to have a genetic origin. Furthermore the capacity for the *de novo *synthesis of long-chain *n*-3 PUFA, DHA and DPA, in muscle was elevated in DUhTP mice fed standard chow compared to DU6 mice. This may lead to increased insulin sensitivity, an association which has been previously observed in human skeletal muscle containing increased proportions of PUFA with twenty and twenty-two carbons [35]. Increased *n*-3 PUFA content in muscle cells was identified as a key driver of inner mitochondrial membrane properties, thereby affecting membrane-associated proteins [36]. Carnitine palmitoyltransferase activity was increased by dietary *n*-3 PUFA suggesting an enhanced lipolytic capacity in skeletal muscle and enhanced mitochondrial membrane fluidity [37]. It is therefore tempting to speculate that the increased *n*-3 PUFA concentrations in muscle may contribute to a metabolic status in DUhTP that leads to the *energy usage phenotype *and to the ability to adapt more successfully to a HFD. In contrast, DU6 mice have significantly less long-chain *n*-3 PUFA concentrations in muscle, they have lower insulin signalling capacity (unpublished results) and probably less oxidative capacity to burn fatty acids in skeletal muscle resulting in the *energy storage phenotype*.

### Different responses to n-3 HFD or n-6 HFD

The mechanisms underlying diet-induced obesity are still not fully understood, but are influenced by a range of genetic factors [38]. The present study investigated metabolic responses to isocaloric high-fat diets enriched with either *n*-3 PUFA or *n*-6 PUFA in two long-term selected lines of mice. The metabolic responses of the animals were defined by measuring key markers of lipid and glucose metabolism in plasma, liver, muscle and adipose tissue and by determining fatty acid composition of liver and muscle. In agreement with many previous studies, fatty acid composition of body tissues was mainly determined by dietary fat intake in both lines of mice. [39,40]. However, the specific metabolic responses in body tissues to a HFD enriched with *n*-3 or *n*-6 PUFA differed markedly between the two selection lines. Major effects are discussed below, separately, for lipid and glucose metabolism. Different responses to HFD occurred despite similar calorie intake per bwt^0.75 ^between the feeding groups and between the selection lines. Thus, our data suggest that mice with a predisposition for obesity development, the *energy storage phenotype*, or to leanness, the *energy usage phenotype*, respond differently to isocaloric HFD rich in *n*-3 PUFA or *n*-6 PUFA. However, the formulation of the diets used in the present study had two limitations. Firstly, differences in protein content of the control diet and the high-fat diets could have potentially influenced some metabolic processes. Secondly, the higher content of myristic acid in *n-3 *HFD may have modified some of the biological effects. Although unlikely, the magnitude of such potential influences could not be addressed in the present study.

### Lipid metabolism

One of the most important contributing factors to obesity development is the increased consumption of energy-dense foods resulting in higher calorie intake and higher intake of dietary fat [41]. Interestingly, only the DU6 mice showed a significant increase in adipose tissue weight in response to both the n-3 HFD and the n-6 HFD. Body weight increased significantly in DU6 mice fed either n-3 or n-6 HFD due to the high calorie density of these diets, although food intake measured in grams was reduced. The higher body weight of DU6 mice was mainly due to increased fat deposition, emphasising the *energy storage phenotype *of these animals. The effects of the HFD on parameters of adiposity were almost missing in DUhTP mice. Interestingly, the increase in adiposity in DU6 mice was not caused by changes in food intake; the calorie intake per bwt^0.75 ^was very similar between the different selection lines. These data clearly underscore the biological importance of genetically determined pathways of energy utilisation that contribute to the development of obesity.

Many dietary components and metabolic conditions are known to be involved in nutrient partitioning that shift energy from storage in adipose tissues to energy utilisation in muscle and to muscle protein synthesis [42-45]. A lower capacity to oxidise fat in skeletal muscle and liver in high fat feeding conditions was stipulated as the main driver of shifting energy into storage as fat in obesity-prone rats [44]. Data from the present study suggest that dietary *n*-3 and *n*-6 PUFA may play a role in nutrient partitioning. The DUhTP mice fed a HFD showed a remarkable adaptive response. Although body weight was increased in the n-3 HFD group, total cholesterol levels were decreased when DUhTP mice were fed n-3 HFD, indicating a health benefit on lipid metabolism of the *n*-3 PUFA diet by inhibiting cholesterol synthesis [46]. In addition, in DUhTP there was only a trend of an increase in adipose tissue weights and a slight, but significant increase in plasma leptin concentrations when fed n-3 HFD, conveying further health benefits on lipid metabolism. These intriguing findings warrant further study and may involve *n*-3 PUFA-mediated changes in peripheral oxidation of fatty acids, especially in individuals with an *energy usage phenotype *[37,47,48].

### Glucose metabolism

The main differences in glucose metabolism responses to n-3 HFD and n-6 HFD for the two selection lines are described by the following main findings. Plasma glucose concentrations were increased by both HFD in DU6 mice. In addition, plasma insulin concentrations in DU6 fed n-6 HFD were increased suggesting development of insulin resistance [49]. In contrast, HFD-induced hyperglycaemia and hyperinsulinaemia was absent in DUhTP mice. Interestingly, in both selection lines n-3 HFD reduced hepatic protein expression of a key component of the insulin signalling pathway, the β-subunit of the IR β, while the PKC ζ signalling protein concentration was reduced in liver tissue of DU6 mice only. In contrast, in abdominal adipose tissue, the PKC ζ protein expression was strongly increased by n-3 HFD indicating a higher capacity for glucose uptake. Importantly, this effect was only observed in DUhTP mice. In previous studies it was suggested that the adipose tissue is a key player in modulating insulin-sensitising effects of dietary *n*-3 PUFA [50,51]. Although the underlying molecular pathways are still uncertain, our data suggest that up-regulation of PKC ζ within the insulin signalling cascade by dietary *n*-3 PUFA may explain the insulin-sensitising effects. The present study showed for the first time that PKC ζ may be a target of *n*-3 PUFA action in a tissue-specific manner. It is increasingly appreciated that the insulin-sensitising effects in adipocytes may be crucial for the health benefits of dietary *n*-3 PUFA [36].

Interestingly, PKC ζ expression was unchanged in adipose tissue when DU6 mice were fed n-3 HFD, but hepatic insulin signalling parameters decreased. Consequently, plasma glucose concentrations increased but without any changes in insulin concentration. It is intriguing that muscle tissue did not respond to the HFD in either of the selection lines suggesting that skeletal muscle may be less responsive to dietary regulating of insulin sensitivity in mice fed HFD. Comparable results were obtained in a study using C57BL/6J mice after an eight-week HFD showing only minor changes in muscular transcriptome [52]. The increase in PKC ζ expression in adipose tissue and the decrease in liver insulin signalling in DUhTP mice offers novel pathways for cellular mechanism of glucose partitioning in response to increased dietary *n*-3 PUFA. This shift in glucose re-partitioning may also be induced in DU6 mice fed n-3 HFD; however, any effect will be less efficient due to the lack of increased PKC ζ expression in adipose tissue. This notion is supported by our observation of increased plasma glucose concentrations with n-3 HFD in DU6 mice. HFD enriched with *n*-6 PUFA led to hyperglycaemia and hyperinsulinaemia in DU6 mice indicating insulin resistance which is commonly induced by HFD feeding [49]. The observed decrease in PKC ζ expression in adipose tissue may contribute to development of insulin resistance in the *energy storage phenotype*, again, demonstrating the important metabolic role of adipose tissue in the regulation of insulin sensitivity. In contrast, DUhTP mice showed only small shifts in hepatic insulin signalling when fed with n-6 HFD which was paralleled by plasma glucose and insulin concentrations within the normal physiological range. The data suggest that the capacity to deposit glucose in insulin-sensitive adipose tissue and liver was maintained in the *energy usage phenotype *even in the face of a n-6 HFD.

### Fatty acid composition

The n-3 and n-6 HFD resulted in different fatty acid composition of liver and muscle in DU6 and DUhTP mice. These changes in fatty acid composition reflect the fatty acid composition of the diet which has been demonstrated previously in studies by Valencak and Ruf [19] and Poureslami et al. [53]. Diet-induced changes were observed in the content of myristic acid which is increased in both, muscle and liver of each line fed n-3 HFD reflecting the high myristic acid content of this diet. Similarly, the increase in *n*-3 PUFA concentrations with a simultaneous decrease of *n*-6 PUFA concentrations in muscle and liver with n-3 HFD feeding was observed in both lines of mice indicating a dominant influence of the dietary fat content. However, some differences seemed to be set by long-term selection of the mice lines indicating a high heritability of fatty acid composition in some tissues. The heritability of body tissues fatty acid composition, especially the deposition of *n*-3 PUFA, has been widely studied in many species, including pigs, salmon and humans [34,54,55]. In DUhTP mice deposition of ALA and EPA was lower in muscle resulting in a smaller sum of *n*-3 PUFA when the n-3 HFD was fed. The lower content of *n*-3 PUFA in muscle, most likely located in cell and mitochondrial membranes, suggests a lower susceptibility to peroxidation compared to DU6 [56], thereby contributing to muscle cell viability and performance in DUhTP. It is tempting to speculate that an increase in the antioxidant capacity in DUhTP mice may also lower susceptibility to peroxidation, thus providing further health benefits of dietary *n*-3 PUFA [57]. This may contribute to the increased running performance in DUhTP mice and support the *energy usage phenotype*. An increase in the *n*-6 PUFA content of body tissues was expected with n-6 HFD feeding, however, this was only observed in DU6 liver and muscle and not in the DUhTP animals indicating again a genetic component of fatty acid deposition. Another remarkable difference in specific fatty acid deposition in muscle was observed regarding the SFA content, especially palmitic and stearic acid. Feeding *n*-3 HFD increased the content of these SFA in DU6 but not in DUhTP mice. These results may suggest that lipogenic pathways are amplified in the DU6 line because palmitic acid is the end product of *de novo *fatty acid synthesis. Enhanced capacity for lipogenesis, even when HFD are fed, may contribute to the development of the *energy storage phenotype *of the DU6 mice.

## Summary

Data presented in this paper advance our understanding about the interactions between genetic predisposition and environmental influences in the development of obesity and its metabolic complications. Mice from long-term selection experiments served as models to examine the contributions of genetic factors in determining specific dietary influences on metabolic regulation relevant to obesity development. Mice lines with distinct biological traits, of an "*energy storage phenotype*", the DU6 mice; and of an "*energy usage phenotype*", the DUhTP mice served to investigate whether different genetic predispositions for obesity or leanness may favour specific metabolic pathways and down-stream effects of lipid or carbohydrate metabolism. The specific metabolic responses in body tissues to a HFD enriched with *n*-3 or *n*-6 PUFA differed markedly between the two selection lines despite similar calorie intake per bwt^0.75 ^between the selection lines. In DUhTP mice, the effects of the HFD on parameters of adiposity such as large fat pads, high plasma cholesterol, triglyceride, glucose and insulin concentrations were almost missing, drawing attention to the biological importance of genetically determined pathways that contribute to the development of obesity. The results of the present study also suggest that dietary *n*-3 PUFA may play an important role in nutrient partitioning. Metabolic health benefits of dietary *n*-3 PUFA may be explained, at least in part, by changes in insulin action and lipid metabolism through re-partitioning of energy from liver and muscle to adipose tissue. The present study showed for the first time that PKC ζ within the insulin signalling cascade may be a target of dietary *n*-3 PUFA action in a tissue-specific manner. The up-regulation of PKC ζ in adipose tissue may explain the insulin-sensitising effects of dietary *n*-3 PUFA. However, important genotype-diet interactions may explain why such diets may have modest effects in some population groups.

## List of abbreviations

DM: dry matter; HFD: high fat diet, IR β: Insulin receptor β; PKC ζ: protein kinase C ζ; PI3K: p85 subunit of the phosphatidyl inositol-3-phosphate kinase; EPA: eicosapentanenoic acid; DHA: docosahexaenoic acid; DPA: docosapentaenoic acid, PUFA: polyunsaturated fatty acids, SCD: Δ^9 ^desaturase, FAS: fatty acid synthase, SREBP-1: sterol regulatory element-binding protein 1, PPAR: peroxisome proliferator-activated receptor; LA: linoleic acid; AA: arachidonic acid; ALA-α: linolenic acid

## Competing interests

The authors declare that they have no competing interests.

## Authors' contributions

KN, DD, BHB and KH participated in the formulation and design of the study. UR and ML participated in the selection breeding to produce the different mice lines, conducted the animal HFD feeding experiment and collected samples. SJ, NT and HB performed Western Blot analyses in liver, muscle (SJ) and adipose tissues (HB) including final analysis of data. KN, FS and DD performed the fatty acid and blood metabolite analyses. GN performed statistical analyses of the data. BHB, KN and KH developed the manuscript in its final form. All authors read and approved the final manuscript.
